# Refractive Changes Following Premature Posterior Capsulotomy Using Neodymium:Yttrium–Aluminum–Garnet Laser

**DOI:** 10.3390/jpm12020272

**Published:** 2022-02-13

**Authors:** Chia-Yi Lee, Tsai-Te Lu, Yaa-Jyuhn James Meir, Kuan-Jen Chen, Chun-Fu Liu, Chao-Min Cheng, Hung-Chi Chen

**Affiliations:** 1 Department of Ophthalmology, Show Chwan Memorial Hospital, Changhua 50093, Taiwan; ao6u.3msn@hotmail.com; 2 Institute of Biomedical Engineering, National Tsing Hua University, Hsinchu 30010, Taiwan; chemchad@gmail.com (T.-T.L.); chaomin@mx.nthu.edu.tw (C.-M.C.); 3 Department of Biomedical Sciences, College of Medicine, Chang Gung University, Linkou 333323, Taiwan; hercats1@gmail.com; 4 Department of Ophthalmology, Chang Gung Memorial Hospital, Linkou Branch, Taoyuan 33305, Taiwan; cgr999@gamil.com; 5 Department of Medicine, College of Medicine, Chang Gung University, Linkou 333323, Taiwan; legendlcf@gmail.com; 6 Department of Ophthalmology, Chang Gung Memorial Hospital, Keelung 20401, Taiwan; 7 Program in Molecular Medicine, National Yang Ming Chiao Tung University, Taipei 112304, Taiwan; 8 Center for Tissue Engineering, Chang Gung Memorial Hospital, Linkou 33305, Taiwan

**Keywords:** Nd:YAG laser capsulotomy, refractive change, hyperopic, posterior capsular opacification

## Abstract

We aimed to determine the timing of neodymium:yttrium–aluminum–garnet (Nd:YAG) laser capsulotomy on corrected-distance visual acuity (CDVA), intraocular pressure (IOP), and spherical equivalent (SE) in patients with posterior capsular opacification (PCO). There were 59 patients with unilateral PCO and a history of Nd:YAG laser capsulotomy enrolled and further divided into the early Nd:YAG group (timing < 12 months, *n* = 25) and late Nd:YAG group (timing > 12 months, *n* = 34) depending on the elapsed months from phacoemulsification to Nd:YAG laser capsulotomy. The primary outcomes were CDVA, IOP, and SE before (immediately before Nd:YAG laser capsulotomy) and after (weeks one and four after the laser treatment). The independent t test was applied to analyze the difference in CDVA, IOP, and SE between the two groups, while the generalized estimating equation with Bonferroni adjustment was conducted to evaluate the effect of all the parameters on the change in SE with adjusted odds ratio (aOR) and 95% confidence interval (CI). The CDVA showed significant improvement in both the early Nd:YAG group (*p* = 0.005) and the late Nd:YAG group (*p* = 0.001), and hyperopic change occurred in both the early Nd:YAG group (*p* = 0.003) and the late Nd:YAG group (*p* = 0.017). The early Nd:YAG group revealed more significant hyperopic change compared with the late Nd:YAG group four weeks after Nd:YAG treatment (*p* < 0.001), which was still significant after multivariable analysis (aOR: 0.899, 95% CI: 0.868–0.930, *p* = 0.011). In addition, a deeper ACD (aOR: 0.764, 95% CI: 0.671–0.869, *p* = 0.019) was significantly correlated with hyperopic change. In conclusion, Nd:YAG laser capsulotomy performed within one year after cataract surgery may lead to significant hyperopic change, in which the ACD alteration affects the hyperopic shift significantly.

## 1. Introduction

Posterior capsule opacification (PCO) is the most common delayed complication of modern cataract surgery [1,2], occurring at the first to fifth years postoperatively in 1.2% to 13.2% of patients [3]. The rate of PCO is different among each type of intraocular lens (IOL) design [4,5]. In spite of decreasing rates of PCO by using advanced IOL biomaterials and optic edge designs [6,7], PCO is gradually intolerable with the introduction of premium IOL [4]. Once it occurs, PCO is now routinely treated by the neodymium:yttrium–aluminum–garnet (Nd:YAG) laser capsulotomy with significant visual improvement in most cases [8,9]. Effective and safe though, laser capsulotomy is not free of complications including transient intraocular pressure (IOP) elevation, hyphema, uveitis, cystoid macular edema, and retinal detachments that occur most frequently in the first few months [8,10,11].

Apart from the aforementioned biological complications, mechanical effects of laser capsulotomy such as pitting of IOL, dislocation of IOL into the vitreous, and shift in the position of IOL have also be reported [8,12]. Displacement of IOL after laser capsulotomy, which might be influenced by the capsulotomy size [13,14], may theoretically change the effective power of IOL and possibly alter the refractive status of the patients. However, most of previous studies failed to show significant change in refraction before and after Nd:YAG laser capsulotomy [15,16,17], except one study showed a hyperopic shift after Nd:YAG laser capsulotomy [18]. Moreover, the size and shape of Nd:YAG laser capsulotomy, the energy used in Nd:YAG laser capsulotomy, and the designs of IOL did not affect the post-laser refractive status according to previous research [19,20,21]. Still, it remains unclear whether the timing of laser capsulotomy plays a pivotal role in the refractive and visual prognosis of patients after Nd:YAG laser capsulotomy. Since the IOL would keep rotating for at least 6 months after cataract surgery [22], an early Nd:YAG laser capsulotomy may lead to instability of lens position and subsequent refraction change that would need evaluation.

Herein, we aimed to investigate the effect of the timing of Nd:YAG laser capsulotomy on visual outcomes, IOP, and refraction that presented as spherical equivalent (SE). Moreover, the effect of other factors such as keratometry (K), anterior chamber depth (ACD), and axial length (AXL) on the change in the aforementioned parameters was also considered.

## 2. Materials and Methods

### 2.1. Subject Selection and Data Collection

A chart review was performed in a tertiary hospital, and patients were enrolled if they (1) received cataract surgery with single-piece foldable hydrophobic acrylic IOL (SN60AT, Alcon, Fort Worth, TX, USA) implantation since the rate of PCO is lesser in such IOL design [7,23], (2) received Nd:YAG laser capsulotomy, and (3) were followed for at least one year. In addition, the following exclusion criteria were applied to erase the influence of certain diseases on visual performance: (1) the diagnosis of prominent vitreoretinal disease throughout the study period such as age-related macular degeneration, diabetic retinopathy, retinal vascular occlusion, retinal detachment, macular hole, macular pucker, vitreous hemorrhage, etc.; (2) the diagnosis of any type of glaucoma throughout the study period, including primary, secondary, open-angle, angle-closure or normal tension glaucoma; (3) any type of corneal surgery throughout the study period, such as penetrating keratoplasty, laser in situ keratomileusis, and photorefractive keratectomy; and (4) the occurrence of posterior capsular rupture during cataract surgery. In addition, the patients were divided into two groups according to the timing of capsulotomy (elapsed months from phacoemulsification to laser capsulotomy), i.e., the early-Nd:YAG (elapsed timing less than 12 months) and the late-Nd:YAG groups (elapsed timing more than 12 months). 

### 2.2. Ophthalmic Examination

All patients were examined before Nd:YAG laser capsulotomy and at 1 and 4 weeks after Nd:YAG laser capsulotomy, and data of routine ophthalmic examinations were obtained via medical documents. The condition of IOL after Nd:YAG laser capsulotomy was evaluated via slit-lamp biomicroscopy. For the visual outcome, the corrected-distance visual acuity (CDVA) was determined from the Snellen chart and calculated as logarithm of minimal angle of resolution (logMAR). Pneumotonometry was used to measure IOP (Topcon c60, Topcon Corp, Tokyo, Japan). Regarding the refraction status, objective refraction was measured using an autorefractometer (Nikon NRK 8000; Inc., Tokyo, Japan) 20 min after instillation of phenylephrine 1% and tropicamide 1%. The SE values were calculated as the sum of the sphere plus half the cylindrical power in diopters (D). Additionally, the K value, ACD, and AXL were measured with an optical biometry (IOL master 500, Zeiss, Oberkochen, Germany). All measurements were repeated three times, and subjects would be excluded if any parameter could not be measured three times in one visit. In addition, the posterior segment examination, such as dilated funduscopic exam, b-scan ultrasonography, or optical coherence tomography, would be arranged if in need. The mean values of CDVA, IOP, SE, K, ACD, and AXL at each visit were enrolled in the analysis.

### 2.3. Statistical Analysis

All the statistical analyses in the current study were performed via the application of SPSS version 20.0 (SPSS Inc., Chicago, IL, USA). Descriptive analysis was applied for the demographic data including age, sex, laterality of eye, energy use in Nd:YAG laser capsulotomy, elapsed time, and ocular parameters between the two groups. The normality of the two groups was tested by Kolmogorov–Smirnov test, in which both groups showed normal distribution (both *p* > 0.05). Then repeated one-way ANOVA was used to evaluate the change in CDVA, IOP, SE, K, ACD, and AXL at different visit times in each group. Then the independent t test was adopted to analyze the difference in CDVA, IOP, and SE between the two groups. In the next step, the generalized estimating equation (GEE) was applied to yield the adjusted odds ratio (aOR) with 95% confidence interval (CI) of the early-Nd:YAG group compared with the late Nd:YAG group concerning hyperopic change after adjusting for the effects of time points (later), age (older), sex (female), laterality (left), energy (more), CDVA (worse), IOP (higher), K (steeper), ACD (deeper), and AXL (longer). Moreover, the effects of demographic data and ocular parameters on hyperopic change were also estimated by GEE analysis. For the analysis with three or more values, the Bonferroni adjustment was adopted to refine the results. The significance level in this study was a *p* value less than 0.05, and the *p* value was depicted as *p* < 0.001 if a *p* value less than 0.001 was yielded due to the format of the statistical software.

## 3. Results

A total of 25 patients were selected in the early Nd:YAG group and another 34 individuals were categorized into the late Nd:YAG group. The mean age was 65.72 ± 8.31 years old in the early Nd:YAG group, which was similar to that in the late Nd:YAG group (67.65 ± 7.90) (*p* = 0.369). The energy of Nd:YAG laser capsulotomy was significantly higher in the early Nd:YAG group compared with the late Nd:YAG group (69.52 ± 15.96 versus 28.31 ± 16.90, *p* < 0.001). On the other hand, the elapsed time between the cataract surgery and Nd:YAG laser capsulotomy was prominently longer in the late Nd:YAG group compared with the early Nd:YAG group (16.03 ± 1.91 versus 9.88 ± 1.36, *p* < 0.001). The demographic data and baseline ophthalmic characters of the two groups are shown in Table 1.

The change in ophthalmic parameters in the early-Nd:YAG group are demonstrated in Table 2. The CDVA was 0.75 ± 0.22 LogMAR before the Nd:YAG laser capsulotomy in the early Nd:YAG group, which progressively improved after the Nd:YAG laser capsulotomy to 0.27 ± 0.07 after one week and 0.25 ± 0.08 after four weeks (*p* = 0.005). In addition, a progressive hyperopic shift was also observed from preoperative SE of −1.02 ± 0.40 D to −0.76 ± 0.30 D and −0.67 ± 0.31 D at one week and four weeks after the Nd:YAG laser capsulotomy (*p* = 0.003). Additionally, the ACD became deeper four weeks after the Nd:YAG laser capsulotomy (*p* = 0.002), but the value of K, IOP, and AXL did not change (all *p* > 0.05). On the other hand, the CDVA of the late Nd:YAG group showed significant improvement one week after the Nd:YAG laser capsulotomy (0.73 ± 0.22 versus 0.28 ± 0.08, *p* = 0.001), and the SE of the late Nd:YAG group also revealed significant hyperopic change before and one week after the Nd:YAG laser capsulotomy (−1.01 ± 0.47 versus −0.88 ± 0.49, *p* = 0.017). Both the CDVA and SE between one and four weeks after the Nd:YAG laser capsulotomy did not alter in the late Nd:YAG group. The ACD illustrated progressive deepening of depth in the late Nd:YAG laser capsulotomy (*p* = 0.009), while the IOP, K, and AXL revealed similar values throughout the study period (all *p* > 0.05) (Table 3).

Regarding the changes in CDVA, IOP, and SE between the two groups, both the CDVA and IOP showed no difference between the two groups at the three time points (all *p* > 0.05). For the refractive status, the SE before the Nd:YAG treatment (*p* = 0.844) and one week after the Nd:YAG treatment (*p* = 0.061) were similar between the two groups. However, the early Nd:YAG group showed a prominent hyperopic change compared with the late Nd:YAG group four weeks after the Nd:YAG treatment (−0.67 ± 0.31 versus −0.85 ± 0.46, *p* < 0.001) (Table 4). In the multivariable analysis, the SE was significantly hyperopic in the early Nd:YAG group compared with that in the late Nd:YAG group after Bonferroni adjustment (aOR: 0.899, 95% CI: 0.868–0.930, *p* = 0.011) (Table 5). Additionally, a later time point (aOR: 0.893, 95% CI: 0.846–0.943, *p* = 0.024) and a deeper ACD (aOR: 0.764, 95% CI: 0.671–0.869, *p* = 0.019) were also significantly correlated with a hyperopic change according to the SE value (Table 5).

## 4. Discussion

Briefly, the current study demonstrated a significant hyperopic change in the early Nd:YAG group compared with that in the late Nd:YAG group after four weeks of follow up after the Nd:YAG laser capsulotomy event. Moreover, the deeper ACD was related to a significant hyperopic change. On the other hand, the CDVA and IOP were similar whether the Nd:YAG laser capsulotomy was performed within or more than one year after cataract surgery.

The SE became more hyperopic significantly in the early Nd:YAG group compared with that in the late Nd:YAG group in the current study. In previous studies, the SE showed similar value after Nd:YAG laser capsulotomy whether in monofocal or multifocal IOL [21,24], and no related factors for the hyperopic or myopic change of SE were found except for the IOL design [19,20,25]. However, those studies did not consider the effect of Nd:YAG laser capsulotomy at different time periods, which may be a critical factor for the refraction since IOL stability is not consistent after the cataract surgery [22]. In addition, the effects of potential confounders were not included in the analysis in those studies. To the best of our knowledge, this is a preliminary study to reveal the relationship between timing of Nd:YAG laser capsulotomy and the hyperopic change after adjusting for multiple confounders. Another finding is that the SE in the early Nd:YAG group kept shifting to hyperopic status, while the hyperopic change only occurred within one week in the late Nd:YAG group. There are two possible mechanistic explanations for the hyperopic shift in the early Nd:YAG group. Firstly, the IOL stability and position may still change within one year [26]; thus, the IOL rotation and tilt may contribute to the change in refraction. In addition, the anterior chamber experienced deepening after the Nd:YAG laser capsulotomy in both the early and late Nd:YAG groups, which may have resulted from posterior IOL displacement after Nd:YAG laser capsulotomy. Compared with the steady deepening in the late Nd:YAG group, the abrupt deepening of ACD four weeks after the Nd:YAG laser capsulotomy in the early Nd:YAG group may indicate a posterior shift of IOL due to poor adhesion between the IOL and posterior capsule. Still, why the ACD became significantly deeper between one week and four weeks after Nd:YAG laser capsulotomy than that in the pre-Nd:YAG laser capsulotomy status remains unknown.

Regarding the anterior segment parameters, the ACD four weeks after the Nd:YAG laser capsulotomy was significantly deeper than that in the pre-Nd:YAG laser capsulotomy status in both groups. The ACD stands for the distance between the center of the anterior corneal epithelium and the anterior crystalline lens capsule in phakic individuals or the distance between the center of the anterior corneal epithelium and the anterior IOL surface in pseudophakic patients [27,28]. Accordingly, the deepening of the anterior chamber may have resulted from the posterior shift or displacement of IOL after the Nd:YAG laser capsulotomy, whether performed within one year or not. The total amount of anterior chamber deepening was similar in both groups, while the change between one week and four weeks was more prominent in the early Nd:YAG group. The etiology for the significant deepening of the anterior chamber is unclear but may be related to the hyperopic shift in the early Nd:YAG group, as discussed above. The K in the early Nd:YAG group became numerically steeper one week after the Nd:YAG laser capsulotomy, while there was no prominent trend of K steepening/flattening in the late Nd:YAG group. To address more details for this issue, nine participants in the early Nd:YAG group experienced a steepened K value one week after Nd:YAG laser capsulotomy, ranging from 0.25 D to 1.00 D. Nevertheless, there was neither subjective visual disturbance nor corneal ectasia recorded in these subjects. The reason for the steepening of K in these patients is unknown, and further study may be needed. Although a steeper K is associated with myopic shift [29], the SE still revealed a hyperopic change in the control group. The possible reason for this may be because an insignificant amount of K steepening led to minimal change in refractive status. The AXL showed minimal change throughout the study period in the two groups, nor did the amount of AXL influence the change in CDVA, IOP, or SE in the two groups. It is reasonable since the gross eyeball structure, including the AXL, could not be altered by Nd:YAG laser capsulotomy. Still, because the AXL is associated with refractive status, it may be appropriate to enter it in the analytic model to allow the model to be more integrated.

For the IOP after Nd:YAG laser capsulotomy, transient IOP elevation is not uncommon [11]. However, the long-term IOP is often within the normal range via the use of anti-glaucomatous medication after Nd:YAG laser capsulotomy [30]. In the current study, the IOP remained similar in the study interval, and the timing of Nd:YAG laser capsulotomy did not influence the fluctuation of IOP. In previous studies, the elevation in IOP after Nd:YAG laser capsulotomy commonly developed within 24 h [31,32]. The first measurement of IOP in the current study was one week after the Nd:YAG laser capsulotomy; thus, the IOP may already have subsided to normal range since topical anti-glaucomatous medication such as brimonidine was routinely prescribed for patients receiving Nd:YAG laser capsulotomy according to previous experience [33]. 

Interestingly, higher laser energy tends to serve as a protective factor for IOP elevation, which is in contrast with the common concept that excessive laser energy would lead to inflammatory response and subsequent ocular hypertension. A possible explanation is that the patient receiving higher laser energy was told to apply anti-inflammatory and anti-glaucoma agents more frequently. Additionally, a significantly higher laser energy was found in the early Nd:YAG group. We speculate that individuals who received Nd:YAG capsulotomy in the early postoperative period experienced a more dense/severe PCO formation compared with those who received Nd:YAG capsulotomy in the late period. Due to the prominent PCO, which may need higher Nd:YAG energy to eradicate [34], the visual disturbance in such patients is more severe, thus warranting early Nd:YAG laser capsulotomy.

The CDVA showed no difference between the two groups, and significant improvement compared with pre-laser status was also observed in both groups. In a previous study that surveyed the visual improvement in those with good visual acuity before Nd:YAG laser capsulotomy, the improvement in visual acuity was still significant [9]. The CDVA before Nd:YAG laser capsulotomy in that study was −0.04 ± 0.04, which was much better than the 0.75 ± 0.22 and 0.73 ± 0.22 in the early Nd:YAG group and late Nd:YAG group in the current study, respectively [9]. Thus, it is reasonable for the patients in current study to reach a significant improvement after eradicating the visual-depriving PCO. Although the CDVA of the early Nd:YAG group kept improving in the study interval, the amount of CDVA was similar between the two groups. This may imply that visual recovery could be achieved within one week after the Nd:YAG laser capsulotomy.

There are some limitations in the current study. First, the retrospective design of the current study could have led to heterogeneity of the study population. Second, the small numbers of the study population may have diminished the power of the current study since we only enrolled one type of single-piece foldable hydrophobic IOL to erase the influence of different IOL types. Additionally, PCO photography was absent; thus, the severity of PCO, which might have led to higher laser energy in the early Nd:YAG group, could not be accessed. Moreover, the data of higher order aberrations were absent since we did not perform such analysis routinely in patients scheduled for Nd:YAG laser capsulotomy. Nevertheless, this may have affected the results of the current study minimally since we analyzed the visual acuity but not visual quality.

## 5. Conclusions

In conclusion, individuals receiving Nd:YAG laser capsulotomy within one year of cataract surgery may experience more hyperopic shift compared with those receiving Nd:YAG laser capsulotomy after a longer time. Furthermore, the alteration of ACD may be correlated with the hyperopic shift in patients with different timing of Nd:YAG laser capsulotomy. Consequently, Nd:YAG laser capsulotomy should be held off until one year after the cataract surgery to avoid IOL instability and subsequent refractive change, and early Nd:YAG laser capsulotomy may account for the abnormal postoperative hyperopic shift. Further large scale prospective studies that evaluate the effect of Nd:YAG laser capsulotomy timing on the refractive status in patients with multifocal IOL implantation are mandatory.

## Figures and Tables

**Table 1:** Basic characters of the study population.

Characters	Early Nd:YAG Group (*n* = 25)	Late Nd:YAG Group (*n* = 34)	*p* Value
Age (year, mean ± SD)	65.72 ± 8.31	67.65 ± 7.90	0.369
Gender (male:female)	11:14	10:24	0.282
Laterality (right:left)	14:11	15:19	0.435
Energy (mJ, mean ± SD)	69.52 ± 15.96	28.31 ± 16.90	<0.001 *
Mean elapsed time (months)	9.88 ± 1.36	16.03 ± 1.91	<0.001 *
Median elapsed time (months)	10	16	-
CDVA	0.75 ± 0.22	0.73 ± 0.22	0.091
IOP (mmHg)	16.22 ± 2.91	15.67 ± 3.47	0.524
SE (diopter)	1.02 ± 0.41	1.01 ± 0.47	0.915
K (diopter)	42.18 ± 0.47	42.28 ± 0.60	0.460
ACD (mm)	2.51 ± 0.13	2.47 ± 0.11	0.180
AXL (mm)	24.27 ± 1.69	23.66 ± 1.48	0.149

Nd:YAG: neodymium:yttrium–aluminum–garnet; N: number; SD: standard deviation; CDVA: corrected distance visual acuity; IOP: intraocular pressure; SE: spherical equivalent; K: keratometry; ACD: anterior chamber depth; AXL: axial length. * denotes significant difference between the two groups.

**Table 2:** Change in parameters in the early Nd:YAG group.

Parameter	Pre-Nd:YAG	Post-Nd:YAG 1 Week	Post-Nd:YAG 4 Weeks	*p* Value	
				Raw	Bonferroni Adjustment
CDVA	0.75 ± 0.22 ^A^	0.27 ± 0.07 ^B^	0.25 ± 0.08 ^C^	<0.001 *	0.005 *
IOP (mmHg)	16.22 ± 2.91	16.15 ± 3.22	16.07 ± 3.29	0.975	1.000
SE (diopter)	−1.02 ± 0.40 ^A^	−0.76 ± 0.30 ^B^	−0.67 ± 0.31 ^C^	<0.001 *	0.003 *
K (diopter)	42.18 ± 0.47 ^B^	42.38 ± 0.47 ^A^	42.53 ± 0.51 ^A^	0.020 *	0.351
ACD (mm)	2.51 ± 0.13 ^B^	2.52 ± 0.13 ^B^	2.60 ± 0.14 ^A^	0.001 *	0.002 *
AXL (mm)	24.27 ± 1.69	24.14 ± 1.33	24.16 ± 1.39	0.682	0.940

Nd:YAG: neodymium:yttrium–aluminum–garnet; CDVA: corrected distance visual acuity; IOP: intraocular pressure; SE: spherical equivalent; K: keratometry; ACD: anterior chamber depth; AXL: axial length. * denotes significant difference among the three periods. ^ABC^: intergroup comparison, the same letter represents no significant difference among groups.

**Table 3:** The change in parameters in the late Nd:YAG group.

Parameter	Pre-Nd:YAG	Post-Nd:YAG 1 Week	Post-Nd:YAG 4 Weeks	*p* Value	
				Raw	Bonferroni Adjustment
CDVA	0.73 ± 0.22 ^A^	0.28 ± 0.08 ^B^	0.28 ± 0.07 ^B^	<0.001 *	0.001 *
IOP (mmHg)	15.67 ± 3.47	15.74 ± 3.44	15.21 ± 3.31	0.774	0.825
SE (diopter)	−1.01 ± 0.47 ^A^	−0.88 ± 0.49 ^B^	−0.85 ± 0.46 ^B^	0.002 *	0.017 *
K (diopter)	42.29 ± 0.10	42.30 ± 0.09	41.70 ± 0.63	0.347	0.956
ACD (mm)	2.47 ± 0.11 ^A^	2.53 ± 0.14 ^B^	2.57 ± 0.15 ^C^	<0.001 *	0.009 *
AXL (mm)	23.66 ± 1.48	23.83 ± 1.47	23.84 ± 1.43	0.439	0.776

Nd:YAG: neodymium:yttrium–aluminum–garnet; CDVA: corrected distance visual acuity; IOP: intraocular pressure; SE: spherical equivalent; K: keratometry; ACD: anterior chamber depth; AXL: axial length. * denotes significant difference among the three periods. ^AB^^C^: intergroup comparison, the same letter represents no significant difference among groups.

**Table 4:** The comparison of vision, intraocular pressure, and refraction between the two groups at different time points.

Parameter	Early Nd:YAG Group	Late Nd:YAG Group	*p* Value
CDVA			
Pre-Nd:YAG	0.75 ± 0.22	0.73 ± 0.22	0.584
Post-Nd:YAG 1 week	0.27 ± 0.07	0.28 ± 0.08	0.881
Post-Nd:YAG 4 weeks	0.25 ± 0.08	0.28 ± 0.07	0.413
IOP			
Pre- Nd:YAG	16.22 ± 2.91	15.67 ± 3.47	0.735
Post-Nd:YAG 1 week	16.15 ± 3.22	15.74 ± 3.44	0.623
Post-Nd:YAG 4 weeks	16.07 ± 3.29	15.21 ± 3.31	0.797
SE			
Pre- Nd:YAG	−1.02 ± 0.40	−1.01 ± 0.47	0.844
Post-Nd:YAG 1 week	−0.76 ± 0.30	−0.88 ± 0.49	0.061
Post-Nd:YAG 4 weeks	−0.67 ± 0.31	−0.85 ± 0.46	<0.001 *

Nd:YAG: neodymium:yttrium–aluminum–garnet; CDVA: corrected distance visual acuity; IOP: intraocular pressure; SE: spherical equivalent. * denotes significant difference between the two groups.

**Table 5:** The effect of different period of Nd:YAG on hyperopic change in spherical equivalent.

Parameter	aOR	95% CI		*p* Value	
		Lower	Upper	Raw	Bonferroni Adjustment
Early Nd:YAG	0.899	0.868	0.930	<0.001 *	0.011 *
Late Nd:YAG (reference)					
Time point (later)	0.893	0.846	0.943	<0.001 *	0.024 *
Age (older)	0.996	0.983	1.008	0.502	0.807
Sex (female)	0.946	0.754	1.189	0.636	0.726
Laterality (left)	0.908	0.752	1.096	0.316	0.616
Energy (higher)	1.003	0.997	1.009	0.342	0.963
CDVA (worse)	1.062	0.913	1.235	0.438	0.928
IOP (higher)	1.002	0.992	1.012	0.693	0.955
K (steeper)	0.987	0.975	0.999	0.030 *	0.330
ACD (deeper)	0.764	0.671	0.869	<0.001 *	0.019 *
AXL (longer)	1.045	0.989	1.104	0.115	0.487

aOR: adjusted odds ratio; CI: confidence interval; Nd:YAG: neodymium:yttrium–aluminum–garnet; CDVA: corrected distance visual acuity; IOP: intraocular pressure; K: keratometry; ACD: anterior chamber depth; AXL: axial length. * denotes significant correlation with hyperopic change.

## Data Availability

The data are available upon reasonable request.
